# Is the Association Between Education and Sympathovagal Balance Mediated by Chronic Stressors?

**DOI:** 10.1007/s12529-021-10027-9

**Published:** 2021-09-27

**Authors:** Benjamin P. van Nieuwenhuizen, Aydin Sekercan, Hanno L. Tan, Marieke T. Blom, Anja Lok, Bert-Jan H. van den Born, Anton E. Kunst, Irene G. M. van Valkengoed

**Affiliations:** 1 Department of Public Health, Amsterdam UMC, University of Amsterdam, Amsterdam, The Netherlands; 2 Department of Surgery, OLVG, Amsterdam, The Netherlands; 3 Department of Clinical and Experimental Cardiology Amsterdam UMC, University of Amsterdam, Amsterdam, The Netherlands; 4 Netherlands Heart Institute, Utrecht, The Netherlands; 5 Department of Psychiatry, Amsterdam UMC, University of Amsterdam, Amsterdam, The Netherlands; 6 Department of Vascular Medicine, Amsterdam UMC, University of Amsterdam, Amsterdam, The Netherlands

**Keywords:** Socioeconomic status, Education, Heart rate variability, Baroreflex sensitivity, Chronic, Stress

## Abstract

**Background:**

This study investigated whether raised chronic stress in low education groups contributes to education differences in cardiovascular disease by altering sympathovagal balance.

**Methods:**

This study included cross-sectional data of 10,202 participants from the multi-ethnic, population-based HELIUS-study. Sympathovagal balance was measured by baroreflex sensitivity (BRS), the standard deviation of the inter-beat interval (SDNN) and the root mean square of successive differences between normal heartbeats (RMSSD). The associations between chronic stressors (work, home, psychiatric, financial, negative life events, lack of job control and perceived discrimination) in a variety of domains and BRS, SDNN and RMSSD were assessed using linear regression, adjusted for age, ethnicity, waist-to-hip ratio and pack-years smoked. Mediation analysis was used to assess the contribution of chronic stress to the association between education and sympathovagal balance.

**Results:**

Modest but significant associations were observed between financial stress and BRS and SDNN in women, but not in RMSSD nor for any outcome measure in men. Women with the highest category of financial stress had 0.55% lower BRS (ms/mmHg; β = -0.055; CI = -0.098, -0.011) and 0.61% lower SDNN (ms; β = -0.061; CI = -0.099, -0.024) than those in the lowest category. Financial stress in women contributed 7.1% to the association between education and BRS, and 13.8% to the association between education and SDNN.

**Conclusion:**

No evidence was found for the hypothesized pathway in which sympathovagal balance is altered by chronic stress, except for a small contribution of financial stress in women.

**Supplementary Information:**

The online version contains supplementary material available at 10.1007/s12529-021-10027-9.

## Introduction

Cardiovascular disease (CVD) morbidity and mortality exhibit stark socioeconomic disparities [1, 2]. While differences in lifestyle factors, such as smoking and physical inactivity, contribute importantly to these disparities, it is incompletely understood which other factors contribute to the observed higher CVD risk in socioeconomically disadvantaged groups [3]. Improved understanding of the risk factors and the mechanisms through which low socioeconomic status (SES) is related with CVD can contribute to novel targets for prevention and treatment of CVD for these high risk populations.

It has been postulated that a part of the SES disparities in CVD result from so called ‘psycho-biological pathways’. Psycho-biological pathways, a term from the psychological literature, are ‘pathways through which psychosocial factors stimulate central nervous system activation of autonomic, neuroendocrine and immunological responses´ [3–5]. Increased chronic psychosocial stress (e.g. work stress, stressful life events or financial stress) is associated with low SES [3, 6, 7]. This is likely to be due to risk factors in the work environment (high demand and low control), physical (e.g. residential crowding) and social (e.g. crime and social resources) factors in the living environment and personal factors (e.g. financial strain) [8]. Stress factors associated with a low SES may lead to CVD (e.g. coronary heart disease), mediated by altered sympathovagal balance [3, 7].

Sympathovagal balance reflects the relative activation of the parasympathetic and sympathetic arms of the autonomic nervous system, which exert homeostatic control over the heart and most other viscera. Short-term fluctuations in either are an adaptive response, eliciting rapid changes in cardiac function in response to an acute change in the environment [9]. However, sustained sympathetic overactivation and/or parasympathetic underactivation is a maladaptive response that is associated with CVD and several of its traditional risk factors including hypertension, diabetes and obesity [4, 10], and that, if present in groups with low SES, may contribute to their elevated CVD burden.

Supporting evidence on this proposed role of sympathovagal balance is derived from studies into its association with low SES. A direct association was demonstrated between low occupation level and decreased heart rate variability (HRV), as measured by the standard deviation of the NN interval (SDNN), low frequency power and high frequency power in a population of male civil servants (n = 2197) [11]. Two studies on smaller study populations from the US showed that low education, occupation and income were associated with decreased SDNN and frequency domain measures [12, 13]. Our group recently observed that low education and occupation level are associated with decreased baroreflex sensitivity (BRS) and decreased SDNN [14]. These results point to a general increase in sympathetic activity and/or decrease in parasympathetic activity in low SES groups.

The potential role of psychosocial stress derives from studies into its association with both CVD risk and SES. Psychosocial stress, both acute and chronic, is a well-established risk factor for CVD morbidity and mortality [15, 16]. Socioeconomic gradients in psychosocial stressors have also been consistently observed [17, 18]. These stressors come in the form of ‘life events, chronic stress, perceived stress, and daily hassles’ [17]. Chronic stress appears to play a more significant role in contributing to the CVD burden than acute stress, although much of the evidence comes from studies specifically on work stress [19].

To provide support for the role of the psycho-biological pathway, it is important to directly study the link between psychosocial stress and sympathovagal balance. Experiments testing autonomic responses to acute stressors appear to show consistent results [20]. For instance, short term changes in HRV are observed during experimental stress tests (e.g. the Stroop color word test), preceding a university examination and a simulation of a medical emergency. However, these types of experimental conditions often do not resemble challenges in the real world [3]. Moreover the association between sympathovagal balance and chronic stress would be more relevant to support the psycho-biological pathway, given the known contribution of chronic stress to the burden of CVD [21]. However, the current evidence for the association between sympathovagal balance and chronic stress is mixed [22–25]. While several studies found differences in HRV parameters by job strain, perceived mental stress during the working day, emotional stress, state-trait anxiety, cumulative stress and effort-reward imbalance [23, 24, 26–38], other studies have also found a variety of HRV parameters that were not associated with various chronic stress domains [23–25, 27, 29–31, 33–38]. This inconsistency may be due to the study of different domains of stress such as effort-reward imbalance (a specific component of work stress) or discrimination [29, 39, 40]. This calls for studies that assess and compare a variety of different chronic stress factors across populations within a single context.

This study investigated whether the association between one SES indicator (education) and sympathovagal balance is mediated via an indirect effect of chronic stressors within a large multi-ethnic population (HELIUS study) from Amsterdam, the Netherlands. As part of this analysis, we first assessed whether there was an association between chronic stress variables and measures of sympathovagal balance.

## Methods

### Population

This study used a cross-sectional design with data from the HEalthy LIfe in an Urban Setting (HELIUS) study, conducted in Amsterdam, the Netherlands. HELIUS has been described in detail elsewhere [41]. In brief, the baseline data collection took place in 2011–2015. Participants, aged 18–70 years and living in Amsterdam, were sampled randomly from the municipality register after stratification by the following ethnic groups: Dutch, Surinamese (2 groups), Ghanaian, Turkish and Moroccan. These are the largest ethnic groups residing in Amsterdam. The HELIUS study was conducted in accordance with the Declaration of Helsinki and has been approved by the Amsterdam UMC, location AMC, Ethical Review Board. All participants provided written informed consent.

A total of 90,019 participants received a written invitation. Approximately 55% were contacted, either by regular mail or after an additional home visit. Out of the 24.789 individuals that agreed to participate in HELIUS, 22,165 had completed both the physical examination and the questionnaire, and A subset of this sample (n = 13,726) received a continuous beat-to-beat registration of blood pressure by finger plethysmography, using the Nexfin device (Edwards Lifesciences, Irvine, CA, USA). This signal was used to conduct HRV analysis.

Participants were excluded who did not have a sinus rhythm, based on 10 s ECG recordings, obtained separately from the photoplethysmography recording used to measure BRS and HRV parameters, to exclude recordings likely to have a substantial number of ectopic beats (n = 169). Photoplethysmography recordings shorter than 180 s after the initial calibration and recordings lacking a continuous recording segment of at least 30 beats without calibration were excluded as shorter recording lengths may affect the accuracy of measurement of HRV parameters (n = 3229) [42]. To remove noise and possible ectopic beats, the interbeat intervals obtained from the photoplethysmography recording were filtered using a local moving median filter with a window length of 9 beats. The beats for which the inter-beat interval differed by more than 25% from the local median inter-beat intervals were removed. Recordings in which more than 20% of the beats were removed, were excluded (n = 79). We further excluded participants with missing data for education (n = 82). As a result, data from 5332 women and 4536 men was analyzed.

### Measures of Sympathovagal Balance

xBRS, SDNN and RMSSD, measured by photoplethysmography, were used as indicators for sympathovagal balance. A high value in either measure indicates low sympathetic activity and/or high vagal activity. Non-invasive continuous finger blood pressure measurements were obtained in supine position after 10 min rest. The cuff was applied on the midphalanx of the middle finger of the left hand. If application to this finger was not possible, the left ring or index finger was used or the right hand. Output analysis was performed using custom written software in Matlab, version 2018b [43]. Estimated BRS was determined using the cross-correlation method (xBRS) [44]. In brief, the cross correlation between 10-s series of systolic blood pressures and interbeat interval (IBI) was computed. The xBRS for each segment was obtained by dividing the standard deviation of the IBI by the standard deviation of the systolic blood pressure. The estimated xBRS was computed in ms/mmHg as the geometric mean over all segments with a significant positive cross correlation (P < 0.05). HRV was obtained in ms as the standard deviation of the filtered IBI dataset (SDNN) and the root mean square of successive differences between normal heartbeats (RMSSD), according to guidelines [45]. Our analyses were limited to two measures of HRV that could be calculated in an automated manner within our large study sample because of the large number of associations to be studied. SDNN and RMSSD were chosen because they are conventional measures of HRV [11, 12, 46].

### Education

Education was chosen as the indicator for SES because it is a reliable and stable indicator for SES [47], and because a previous study found that the associations in sympathovagal balance were stronger for education than occupation, while being in the same direction [14]. Information on education was obtained by questionnaire. Education was based on the highest qualification attained, either in the Netherlands or in the country of origin, and was categorized into four groups: (1) never been to school or elementary schooling only, (2) lower vocational schooling or lower secondary schooling, (3) intermediate vocational schooling or intermediate/secondary schooling, or (4) higher vocational schooling or university.

### Chronic Stress Variables

All stress variables were assessed by self-report from a questionnaire administered at baseline. Home stress was defined as perceived psychosocial stress (feeling irritable, anxiety or trouble sleeping within the last 12 months) arising from one's living circumstances at home (never, some of the time, several periods, permanent) [15]. Work stress was defined as perceived psychosocial stress (feeling irritable, anxiety or trouble sleeping within the last 12 months) arising from one's circumstances at work (never, some of the time, several periods, permanent) [15]. Any stressful life events, measured with Nemesis-II questionnaire were operationalized as any type of self-reported threatening experience (e.g. the death of a close relative or friend, major financial crisis) during the previous 12 months (yes/no) [48]. Financial stress was based on answers to the question “Do you have trouble to make ends meet”: no, careful, some difficulty, great difficulty [49]. Lack of job control was quantified with a score ranging between 0 and 100 with a nine item scale which assesses perceived work-related recovery opportunities [50]. Lack of job control of those who had responded that they were not part of the workforce (due to being a house husband/wife, injury etc.), were classified as not applicable. Perceived discrimination was a binary variable assessed by a modified version of the everyday discrimination scale, which has been previously described [51]. Perceived discrimination was marked as present if any of the 9 items were scored with four or more points out of a possible five.

### Ethnicity

A participant was defined as belonging to the Dutch (majority) ethnic group if the participant was born in the Netherlands and whose parents were born in the Netherlands. A participant was defined as one of the ethnic minority groups if the participant was born in the specified country and had at least one parent that was born in the same country (first generation), or was born in the Netherlands but both parents were born abroad (second generation). After data collection, Surinamese subgroups were further classified according to self-reported ethnic origin (South-Asian or African).

### Behavioral Determinants of Sympathovagal Balance

Two variables which are known determinants of sympathovagal balance were conditioned for: smoking and waist-to-hip ratio. Waist-to-hip ratio was defined as waist circumference (in m) divided by hip circumference (in m). Waist circumference was measured using a tape measure at the midway between the lowest rib margin and the iliac crest and hip circumference was measured at the widest point over the trochanter major. Smoking was defined by pack-years smoked. The number of pack-years of smoking was calculated by multiplying the daily smoking rate counted by 20 cigarette containing packs (or equivalent rates for cigars and pipe tobacco) by the number of years smoked. Rolling tobacco and cigar use were converted into an equivalent number of cigarettes.

### Analysis

Baseline characteristics of women and men were provided as means and standard deviations for continuous variables or percentages for categorical variables. Normality was checked in the outcome measures (xBRS, SDNN and RMSSD) with visual inspection, and calculation of skewness and kurtosis. Given a lognormal distribution in xBRS, SDNN and RMSSD, these outcome measures were transformed using the natural logarithm, hereafter referred to as lnBRS, lnSDNN and lnRMSSD.

The associations between chronic stress and sympathovagal balance were assessed with multivariate linear regression models. Separate models were created for each stress variable (exposure) and either lnBRS, lnSDNN, or lnRMSSD (outcome), adjusting for age, ethnicity, waist-to-hip ratio and pack- years smoked. Education was entered into the models as cumulative ranks. To calculate the cumulative ranks, first the proportions of each education category were calculated. The cumulative ranks for the education groups are half of their proportion within the study population plus the sum of the proportions of lower education groups. The regression coefficient resulting from this is also known as the relative index of inequality (RII) and can range from 0 (low education) to 1 (high education) [14, 52, 53]. We performed the analyses separately in women and men because of an observed stronger financial stress gradient women compared to men for lnSDNN (p = 0.024), for lnRMSSD (p = 0.06) and for BRS (p = 0.12), although this was not statistically significant in the latter two cases.

For mediation, the R package ‘LAVAAN’ was used [54]. The calculation of the indirect and total effects, in the context of a structural equation modeling framework, has been described in detail elsewhere [55]. In brief, the total effect is the entire association between independent variable, in this case education, and the dependent variable, lnBRS, lnSDNN or lnRMSSD. The total effect can be split into two paths; one path that runs via a mediating variable is termed the ‘indirect effect’, the other path which links the independent and dependent variables directly is termed the ‘direct effect’. To estimate the indirect and total effects, two models were created for each analysis. (1) a “mediator model”, with the mediator (stressor) as the dependent variable and education as the independent variable, adjusting for age and ethnicity. It was decided a priori that only stressors found to have a significant association with sympathovagal balance measures were entered into mediation models. And an “outcome model”, with the outcome (lnBRS, lnSDNN or lnRMSSD) as the dependent variable and education as the independent variable, adjusting for the mediator, age and ethnicity. The indirect effect is then calculated by multiplying the β coefficient for education from the mediator model (a) and the β coefficient of the mediator from the outcome model (b), i.e. the product of coefficients method. Confidence intervals for the indirect effect were calculated The direct effect (c’) is the β coefficient for education in the outcome model. The total effect (C) is the sum of the direct effect (c’) and the indirect effect (a*b). Thus, to calculate the proportion of the association that is mediated, the indirect effect is divided by the total effect (a*b/(a*b + c’)). Education was entered into the models the same way as described for the multivariate models. Bootstrap standard errors for each regression coefficient were calculated with 5000 draws.

Post hoc sensitivity analyses were performed, wherein the final models were repeated in the population, excluding individuals taking antihypertensive medication (Ca^2+^ channel blockers, β blockers and renin angiotensin system inhibitors), due to the known influence of these medications on sympathovagal balance. Another sensitivity analysis was used to check whether our results differed after converting each financial stress category into dummy variables because the mediation analyses assumed ordinal categories in the mediators.

## Results

Descriptive statistics of the study population, stratified by sex, can be found in table 1. More than half of the women (60%) and men (58%) with the lowest level of education were in the two highest levels of financial stress (Supplemental table 1). With each higher level of education there was a lower proportion of women and men in the two higher financial stress categories, reaching 23% of women and 19% of men with the highest education. A similar although less prominent shift in the distribution of home stress categories occurred across levels of education. Both the lowest and the highest categories of work stress were less common in women and men with successively higher levels of education. For example, 60% of women with the lowest level of education never experienced work stress, while 26% of women with the highest level of education never experienced work stress. Median lack of job control was highest in women (48.1; IQR = 22.2) and men (48.1; IQR = 29.6) in the lowest education level and lowest in women (33.3; IQR = 33.3) and men (25.9; IQR = 25.9) in the highest education level. Perceived discrimination diminished from 28% in women and 35% in men in the lowest education to 18% in women and 15% in men in the highest education level. The proportion of women having suffered a negative life event was lower in higher education levels, while in men the proportion rose with higher education level.

Experiencing work stress was not strongly associated with lnBRS, lnSDNN or lnRMSSD compared to experiencing no work stress (Table 2). Only men sometimes experiencing work stress showed a statistically significant rise of 0.51% in lnBRS compared to men who did not experience work stress. Similarly, no large differences were observed between categories of experienced home stress. Women that experienced multiple periods of home stress had an estimated 0.46% lower lnBRS than women that did not experience home stress. Men who experienced some home stress had an estimated rise of 0.36% in lnBRS and lnSDNN compared to men that did not experience any home stress. There was no consistent pattern in the direction or size of the estimates for work stress or home stress. There was no large or significant differences found in lnBRS or lnSDNN with the presence of negative life events, lack of job control or perceived discrimination.

A clear pattern of decreasing lnBRS and lnSDNN with each higher category of financial stress was evident in women. This ranged from a decrease of 0.19% in lnBRS in the second lowest financial stress category to a 0.55% decrease of lnBRS in the highest financial stress category. The estimate for the highest category of financial stress and BRS reached statistical significance (p = 0.014). For lnSDNN, the estimates ranged from a decrease of 0.3% in the second lowest category of financial stress to 0.61% in the highest category of financial stress. This pattern was not observed for lnRMSSD, in women. In men, no large differences in lnBRS, lnSDNN or lnRMSSD were observed between financial stress categories (Table 3). There was also no consistent pattern in the direction and size of these estimates and neither were any of the estimates found to be statistically significant in men.

The geometric means of xBRS, SDNN and RMSSD were higher in successively higher categories of education level (Table 4). For example, xBRS ranged from 9.5 ms/mmHg (SD = 6.1) in the lowest education level to 17 ms/mmHg in the highest education level in women and from 9.7 (SD = 5.6) in the lowest education level to 14.5 (SD = 9) in the highest education level in men. This pattern was less pronounced for RMSSD, ranging from 0.034 (SD = 1.672) in the lowest education level to 0.046 ms (SD = 1.756) in the highest education level in women. A similar range was observed in men. However, the highest category for education had a slightly lower RMSSD than the intermediate level.

The associations between education and lnBRS and lnSDNN were significant for women and men (table 5). For example, the total effect for lnBRS was β = 0.147 (CI: 0.091, 0.204, p =  < 0.001) in women. Financial stress contributed to this association in women for lnBRS and lnSDNN, but not for lnRMSSD. The indirect effect of financial stress approached statistical significance for lnBRS (β = 0.010; CI: -0.002, 0.023, p = 0.098) and was statistically significant for lnSDNN (β = 0.015; CI: 0.004, 0.027, p = 0.009) for women. Financial stress did not contribute to the association between education and lnBRS or lnSDNN in men. This resulted in a percentage mediated by financial stress of 7.1% in lnBRS and 13.8% in lnSDNN in women. Financial stress did not contribute to the association between education and lnBRS, lnSDNN or lnRMSSD in men.

### Post Hoc Sensitivity Analyses

The analyses repeated after excluding those taking antihypertensive medication did not change the results substantially. In women, the estimate for the association between education and lnBRS via financial stress (indirect effect) was attenuated from 0.01 to 0.006, while the estimate remained similar for lnSDNN (Electronic Supplemental Material 2. Comparing each category of the stress variables to a reference category (lowest level) also produced similar results to the initial models (Electronic Supplemental Material 3 and 4). Furthermore, post hoc, a quadratic term for the stressor was added to each model due to the observation that the distribution of each stressor (except financial stress) was not linear across education level categories. These models produced nearly identical results compared to the initial models, which assumed linearity (data not shown).

## Discussion

### Key Findings

This study investigated the association between chronic stress and sympathovagal balance and the contribution of chronic stress to the association between education and sympathovagal balance. An association was only observed for financial stress and only in women. Financial stress played a modest mediating role in the relationship between education and sympathovagal balance (measured by BRS and SDNN) in women. This contribution could not be demonstrated in men or for RMSSD in either sex.

### Discussion of the Key Findings

Our finding of a modest decrease in BRS and SDNN with higher financial stress may indicate that stress within the financial domain has a specific effect on the autonomic nervous system. The lack of association we found for RMSSD, may indicate this is not the case for vagal activity specifically [56]. This is in line with the finding that financial stress has been shown to have a strong association to neuroendocrine markers of the sympatho-adrenal-medullary and hypothalamic pituitary adrenal axes [57]. Financial stress may reflect the stress resulting from limited material resources which may increase the likelihood of living in an environment filled with higher cardiovascular risk factors (e.g. noise pollution, air pollution, lack of green spaces and high crime rate) [58]. Financial stress may also inhibit health seeking behavior and it may reflect a general lack of control over every day decisions in life, which is associated with a raised cardiovascular risk profile [59, 60].

The association between financial stress and sympathovagal balance (xBRS and SDNN) was found in women, while no association was found in men. The finding of an association between financial stress and sympathovagal balance agrees with previous findings of an association between financial stress and CVD events [61]. It is unclear why this association is different in women and men. It is known that women can experience stress differently because of the ‘dual occupancy’ of care-taking and bread-winning roles and their generally greater role in household management [62]. There is also evidence for different responses of the sympathetic and parasympathetic nervous systems in women and men to physical stimuli and stressors [63]. Financial stress also implicitly contains information on income and/or wealth. It is possible that financial stress corresponds proportionally differently to material financial means in women and men, leading to a different association between financial stress and sympathovagal balance.

It should be remarked that the effect size for the associations between financial stress and sympathovagal balance was small, with for example a reduction of 0.06 ms in the SDNN in the highest financial stress group compared to those with the lowest level of financial stress. This has been indicated to correspond with a 0.06% increase in risk for CVD events and thus may not be of much clinical relevance [10]. Similarly, financial stress explained only a small proportion of the associations between education and BRS (0.07) and SDNN (0.14). Thus financial stress appears not to contribute greatly to the relationship between education and sympathovagal balance.

The other stressors did not appear to contribute to education gradients in sympathovagal balance. This appeared to be due to a lack of association between the stressors and HRV (Table 2). While financial stress was based on a relatively objective question, the lack of association between the other stressors and sympathovagal balance may have been due to their more subjective assessment. Similarly, previous studies using objective measures into specific aspects underlying stress, such as the effort-reward imbalance may have been more likely to observe associations [20]. The results of previous studies are however mixed. While SDNN has been shown to differ by job control in the Whitehall study, no association was found in three other studies conducted in homogeneous worker populations [23, 25, 64]. Several studies have found an association between work related stress and other HRV parameters. Clays et al. found associations between work stress and a time domain measure of HRV (pNN50) and frequency measures of HRV [65]. Effort-reward imbalance has been found to be associated with the root mean squared of successive differences between heart beats, the proportion of heart beat intervals that are over 50 ms long and the ratio between high frequency and low frequency power [28, 29]. However, data on more specific aspects related to stress was not available. A possible explanation of for these mixed findings may be due to differing levels of autonomic desensitization to stress [66].

The pathophysiological consequences of chronic stress may be mediated by a physiological mechanism other than the autonomic nervous system. The hypothalamic–pituitary–adrenal axis may play a more instrumental role in mediating the biological consequences of chronic stress. This system, regulating the timing and quantity of glucocorticoid release has been shown to be dysregulated in chronic stress [67, 68]. It has also been observed that the hypothalamic–pituitary–adrenal axis is implicated with long term and short term physiological stress responses, appraised as challenges, while involvement of the autonomic nervous system may be limited to the short term stressors appraised as threats [69, 70]. Thus, the hypothalamic–pituitary–adrenal axis may be implicated as a more important mediator in the association between chronic stress and CVD morbidity and mortality.

### Limitations

The selection of participants for which the Nexfin recording was of sufficient quality to be included in this study, may have influenced our results. However, characteristics of the study population (ethnicity, sex, education level and chronic stress) did not differ substantially between the entire HELIUS study population and the study population for the current analyses [14]. The low response rate in HELIUS may have affected our findings. Given that individuals with high chronic stress may have been less likely to choose to take part in the study, associations may have been missed due to the underrepresentation of the highest levels of chronic stressors in the study sample [41].

The methods used to define our outcomes may have been suboptimal. Our 5-min Nexfin recording differs from the recommended 24 h electrocardiogram recordings for HRV analysis[46]. However, results from recordings as short as 30 s have been shown be highly correlated with results from 24 h recordings [71]. Photoplethysmography has been shown to correlate well with ECG for measurement of HRV in several studies [72, 73]. However, low frequency fluctuations in inter-beat intervals, such as the very low frequency power, is not possible with short recording lengths [56]. Thus, future studies, using longer recordings may be able to investigate whether education/SES and/or chronic stress differences exist in low frequencies of HRV. Moreover, longer recordings may be used to study the relationship between intra-subject variations in measures of HRV and stress. The gold-standard for BRS recordings is the response measured after administration of phenylephrine. The spontaneously generated xBRS signals used in this study, however, correspond well with pharmacologically induced BRS measures [44].

Potential measurement issues with the mediators may have occurred. Measures of chronic stress may fall victim to recall bias, social desirability or simply misunderstanding the question [74]. Furthermore, several of the stressors (work, home, financial) were assessed by one questionnaire item. Most stress measures (home, work, discrimination, job control, negative life events) have been shown to be associated with negative health outcomes in previous studies and have been adapted from validated questionnaires. However, it is not clear whether the instruments are valid in the specific subgroups in our multi-ethnic study population.

Our choice of carrying out mediation analysis within a structural equation modelling package in R (“LAVAAN”) may have influenced our results. This method limited the format of the mediator to being an ordinal categorical variable, which assumes a linear, continuous relation between categories with the same magnitude of difference between any two adjacent categories. This assumption was checked by conducting two sensitivity analyses, the results of which did not substantially change our findings.

Cross-sectional data is often limited by the inferences that can be made regarding the direction of the associations found. However, education level is by definition defined far before our outcome measures were obtained in the vast majority of individuals. Thus, the question of directionality is likely not hampered by the cross-sectional design of the current study.

## Conclusion

This study found no consistent evidence for a mediating role of chronic stress in the association between education and sympathovagal balance. An association was not observed between sympathovagal balance and most stressors, except for financial stress. This may indicate that psycho-biological pathways involving other physiological systems than the autonomic nervous system mediate socioeconomic differences in the risk of cardiovascular disease via chronic stress.

**Table 1:** Characteristics of the study population

		**Women (5332)**	**Men (4536)**
Age (mean, SD)		43 (13.1)	45 (13.0)
Ethnicity (n, %)	Dutch	938 (17.6)	1083 (23.9)
	South Asian Surinamese	635 (11.9)	668 (14.7)
	African Surinamese	1174 (22.0)	826 (18.2)
	Ghanaian	660 (12.4)	511 (11.3)
	Turkish	870 (16.3)	789 (17.4)
	Moroccan	1055 (19.8)	659 (14.5)
Education	Elementary	1189 (22.3)	587 (12.9)
level (n, %)	Lower	1317 (24.7)	1360 (30.0)
	Intermediate	1504 (28.2)	1357 (29.9)
	Higher	1322 (24.8)	1232 (27.2)
Work stress (n, %)	Never	1729 (40.1)	1777 (44.2)
	Some of the time	1696 (39.3)	1563 (38.9)
	Several periods	579 (13.4)	459 (11.4)
	permanent	310 (7.2)	219 (5.5)
	Not applicable	995	499
	Missing	23	19
Home stress (n, %)	Never	2382 (44.7)	2582 (56.9)
	Some of the time	1973 (37.0)	1432 (31.6)
	Several periods	703 (13.2)	347 (7.6)
	permanent	274 (5.1)	175 (3.9)
Financial stress	No	1268 (23.8)	1399 (30.8)
(n, %)	Careful	1773 (33.3)	1459 (32.2)
	Some difficulty	1417 (26.6)	1049 (23.1)
	Great difficulty	874 (16.4)	629 (13.9)
Experienced negative life event (n, %)		3600 (67.5)	2932 (64.6)
Lack of job control (median, IQR)		59 (29.6)	63 (29.6)
Perceived discrimination (n, %)		1261 (23.6)	1192 (26.3)
Antihypertensive medication (n, %)		961 (18.0)	664 (14.6)
Recording parameters	Recording length (min)	303.5 (42.6)	299.1 (38.2)
(Mean, SD)	Number of included beats	328.2 (62.0)	307.9 (61.2)
Outcome measures	BRS (mmHg)	11.0 (10.5)	10.9 (8.9)
(Median, IQR)	SDNN (ms)	45.5 (29.4)	48.5 (29.7)
	RMSSD (ms)	0.040 (0.033)	0.038 (0.030)

^a^β blockers, calcium channel blockers and renin-angiotensin system blockers

^b^BRS, baroreflex sensitivity

^c^SDNN, standard deviation of the N-N interval

^d^RMSSD, the root mean square of successive differences between normal heartbeats

**Table 2:** The association between chronic stressors and BRS, SDNN and RMSSD, adjusted for age, ethnicity waist to hip ratio and pack-years smoked in women

			**Women**				
		BRS^a^		SDNN^b^		RMSSD^c^	
		RII^d^ (CI^e^)	p value	RII (CI)	p value	RII (CI)	p value
Work stress	Never	ref		ref		ref	
	Some of the time	0.019 (-0.024, 0.062)	0.382	0.026 (-0.011, 0.063)	0.168	0.034 (-0.009, 0.077)	0.123
	Several periods	-0.002 (-0.061, 0.058)	0.952	0.008 (-0.043, 0.059)	0.766	-0.002 (-0.0061, 0.057)	0.955
	permanent	0.000 (-0.084, 0.084)	0.998	0.045 (-0.027, 0.118)	0.223	0.039 (-0.044, 0.123)	0.356
Home stress	Never	ref		Ref			
	Some of the time	-0.007 (-0.037, 0.022)	0.617	-0.006 (-0.031, 0.020)	0.661	-0.007 (-0.036, 0.023)	0.665
	Several periods	-0.046 (-0.087, 0.004)	0.031	-0.018 (-0.054, 0.017)	0.315	-0.015 (-0.057, 0.026)	0.467
	permanent	0.005 (-0.056, 0.067)	0.862	0.016 (-0.037, 0.069)	0.552	0.017 (-0.045, 0.080)	0.585
Financial stress	No	ref				ref	
	Careful	-0.019 (-0.055, 0.016)	0.281	-0.030 (-0.061, 0. 000)	0.052	-0.024 (-0.059, 0.012)	0.197
	Some difficulty	-0.030 (-0.068, 0.008)	0.125	-0.047 (-0.080, -0.014)	0.005	0.034 (-0.009, 0.077)	0.264
	Great difficulty	-0.055 (-0.098,-0.011)	0.014	-0.061 (-0.099, -0.024)	0.001	-0.044 (-0.088, 0.000)	0.052
Negative life event		0.003 (-0.025, 0.031)	0.848	0.003 (-0.021, 0.028)	0.788	0.005 (-0.023, 0.033)	0.731
Job control		0.000 (-0.001, 0.001)	0.475	0.000 (-0.001, 0.001)	0.789	0.000 (0.000, 0.001)	0.332
Discriminations		-0.006 (-0.038, 0.026)	0.705	0.012 (-0.015, 0.040)	0.372	0.005 (-0.027, 0.037)	0.756

^a^BRS, baroreflex sensitivity

^b^SDNN, standard deviation of the N-N interval

^c^RMSSD, the root mean square of successive differences between normal heartbeats

^d^RII, relative index of inequality

^e^CI, confidence interval

**Table 3:** The association between chronic stressors and BRS, SDNN and RMSSD, adjusted for age, ethnicity waist to hip ratio and pack-years smoked in men

			**Men**				
		BRS^a^		SDNN^b^		RMSSD^c^	
		RII^d^ (CI^e^)	p value	RII (CI)	p value	RII (CI)	p value
Work stress	Never	ref		ref		ref	
	Some of the time	0.051 (0.013, 0.088)	0.008	0.025 (-0.007, 0.056)	0.123	0.028 (-0.010, 0.066)	0.146
	Several periods	0.018 (-0.040, 0.077)	0.534	-0.013 (-0.062, 0.036)	0.609	-0.027 (-0.087, 0.032)	0.367
	permanent	-0.027 (-0.115, 0.061)	0.550	0.005 (-0.069, 0.079)	0.893	-0.016 (-0.106, 0.074)	0.727
Home stress	Never	ref		ref		ref	
	Some of the time	0.036 (0.005, 0.067)	0.023	0.036 (0.009, 0.063)	0.010	0.036 (0.004, 0.069)	0.028
	Several periods	-0.049 (-0.103, 0.005)	0.076	-0.021 (-0.068, 0.027)	0.390	-0.005 (-0.062, 0.051)	0.857
	permanent	-0.035 (-0.109, 0.039)	0.356	-0.008 (-0.073, 0.057)	0.808	-0.010 (-0.088, 0.067)	0.792
Financial stress	No	ref		ref		ref	
	Careful	0.013 (-0.023, 0.048)	0.484	0.009 (-0.022, 0.039)	0.584	0.033 (-0.004, 0.070)	0.077
	Some difficulty	0.006 (-0.033, 0.046)	0.763	0.005 (-0.030, 0.039)	0.797	0.033 (-0.008, 0.075)	0.113
	Great difficulty	-0.035 (-0.083, 0.012)	0.148	-0.032 (-0.074, 0.009)	0.129	-0.005 (-0.054, 0.045)	0.854
Negative life event		0.008 (-0.021, 0.038)	0.576	-0.001 (-0.027, 0.024)	0.917	0.016 (-0.015, 0.047)	0.307
Job control		0.000 (-0.001, 0.001)	0.615	0.000 (-0.001, 0.001)	0.840	0.000 (0.000, 0.001)	0.329
Discrimination		-0.012 (-0.045, 0.022)	0.490	0.010 (-0.019, 0.039)	0.497	0.007 (-0.029, 0.042)	0.717

^a^BRS, baroreflex sensitivity

^b^SDNN, standard deviation of the N-N interval

^c^RMSSD, the root mean square of successive differences between normal heartbeats

^d^RII, relative index of inequality

^e^CI, confidence interval

**Table 4:** Geometric (back transformed) means of BRS, SDNN and RMSSD across education level categories in women and men

			**Education level**			
			Elementary	Lower	Intermediate	Higher
Women	BRS^a^ (ms/mmHg)		8.1 (1.8)	10.4 (1.8)	13.2 (1.9)	14.0 (1.9)
	SDNN^b^ (ms)		38.2 (1.5)	44.4 (1.6)	49.2 (1.6)	52.9 (1.6)
	RMSSD^c^ (ms)		0.034 (1.672)	0.040 (1.742)	0.045 (1.732)	0.046 (1.756)
Men	BRS (ms/mmHg)		8.3 (1.7)	9.7 (1.8)	12.2 (1.8)	12.2 (1.8)
	SDNN (ms)		41.3 (1.6)	43.3 (1.6)	51.5 (1.6)	52.5 (1.6)
	RMSSD (ms)		0.033 (1.699)	0.035 (1.723)	0.042 (1.768)	0.040 (1.759)

Given as mean (SD)

^a^SDNN, standard deviation of the N-N interval

^b^BRS, baroreflex sensitivity

^c^RMSSD, the root mean square of successive differences between normal heartbeats

**Table 5:** Contribution of financial stress to the relative index of inequality for education on lnBRS, lnSDNN and lnRMSSD, stratified by sex

		**Indirect effect**		**Total effect**		**Proportion mediated**	
		β (CI^a^)	p value	β (CI)	p value	Proportion^b^ (CI)	p value
Women	lnBRS^c^	0.010 (-0.002, 0.023)	0.096	0.147 (0.088, 0.205)	<0.001	0.071 (-0.014, 0.179)	0.147
	lnSDNN^d^	0.015 (0.004, 0.026)	0.007	0.110 (0.059, 0.161)	<0.001	0.138 (0.035, 0.324)	0.062
	lnRMSSD^e^	0.011 (-0.002, 0.024)	0.099	0.044 (-0.016, 0.101)	0.143	0.243 (-1.680, 2.576)	0.996
Men	lnBRS	0.002 (-0.012, 0.015)	0.755	0.114 (0.057, 0.170)	<0.001	0.018 (-0.115, 0.150)	0.803
	lnSDNN	0.002 (-0.010, 0.014)	0.765	0.103 (0.051, 0.157)	<0.001	0.017 (-0.103, 0.157)	0.792
	lnRMSSD	-0.004 (-0.018, 0.010)	0.562	0.007 (-0.053, 0.068)	0.832	-0.629 (-3.418, 3.863)	0.999

^a^CI, confidence interval

^b^Proportion mediated is the indirect effect (*β*) divided by the total effect (*β*)

^c^SDNN, standard deviation of the N-N interval

^d^BRS, baroreflex sensitivity

^e^RMSSD, the root mean square of successive differences between normal heartbeats

## Supplementary Information

Below is the link to the electronic supplementary material.

Supplementary file1 (DOCX 34 KB)
