# Supplementary material for: Is the Association Between Education and Sympathovagal Balance Mediated by Chronic Stressors?
Source: Int J Behav Med. 2021 Sep 27;29(4):426–37. doi: 10.1007/s12529-021-10027-9 (PMC9338002; doi:10.1007/s12529-021-10027-9)
Supplement: Supplementary file 1 — Supplementary file1 (DOCX 34 KB) [file 12529_2021_10027_MOESM1_ESM.docx]

**Electronic Supplementary Material**

**Electronic Supplementary Material 1 (table).** Characteristics of the study population by education level

|  |  | **Women (5332)** | | | | **Men (4536)** | | | |
| --- | --- | --- | --- | --- | --- | --- | --- | --- | --- |
|  |  | **Education level (n)** | | | | **Education level (n)** | | | |
|  |  | Elementary (1189) | Lower (1317) | Intermediate (1504) | Higher (1322) | Elementary (587) | Lower (1360) | Intermediate (1357) | Higher (1232) |
| **Age** years mean (SD) | | 49.7 (9.6) | 46.3 (12.6) | 38.6 (13.3) | 40.6 (12.9) | 50.0 (11.3) | 47.6 (11.8) | 40.3 (13.6) | 43.7 (12.7) |
| **Financial stress** | None | 161 (13.5) | 296 (22.5) | 299 (19.9) | 512 (38.7) | 123 (21.0) | 293 (21.5) | 406 (29.9) | 577 (46.8) |
| n (%) | Careful | 314 (26.4) | 382 (29.0) | 573 (38.1) | 504 (38.1) | 125 (21.3) | 418 (30.7) | 491 (36.2) | 425 (34.5) |
|  | Some difficulty | 398 (33.5) | 382 (29.0) | 407 (27.1) | 230 (17.4) | 188 (32.0) | 380 (27.9) | 312 (23.0) | 169 (13.7) |
|  | Great Difficulty | 316 (26.6) | 257 (19.5) | 225 (15.0) | 76 (5.7) | 151 (25.7) | 269 (19.8) | 148 (10.9) | 61 (5.0) |
| **Homestress** | Never | 560 (46.2) | 583 (42.6) | 634 (43.0) | 605 (84.4) | 320 (54.5) | 752 (55.3) | 778 (59.3) | 732 (69.1) |
| n (%) | Some of the time | 389 (32.1) | 497 (36.3) | 600 (40.7) | 487 (67.9) | 157 (26.7) | 444 (32.6) | 424 (32.3) | 407 (38.4) |
|  | Several periods | 136 (11.2) | 172 (12.6) | 199 (13.5) | 196 (27.3) | 63 (10.7) | 105 (7.7) | 109 (8.3) | 70 (6.6) |
|  | permanent | 104 (8.6) | 65 (4.8) | 71 (4.8) | 34 (4.7) | 47 (8.0) | 59 (4.3) | 46 (3.5) | 23 (2.2) |
| **Workstress** | Never | 489 (59.8) | 468 (46.1) | 453 (35.8) | 319 (26.2) | 235 (50.8) | 561 (48.7) | 587 (47.7) | 394 (33.6) |
| n (%) | Some of the time | 202 (24.7) | 360 (35.5) | 527 (41.7) | 607 (49.9) | 134 (28.9) | 402 (34.9) | 459 (37.3) | 568 (48.5) |
|  | Several periods | 70 (8.6) | 101 (10.0) | 186 (14.7) | 222 (18.3) | 51 (11.0) | 105 (9.1) | 132 (10.7) | 171 (14.6) |
|  | permanent | 57 (7.0) | 86 (8.5) | 99 (7.8) | 68 (5.6) | 43 (9.3) | 84 (7.3) | 53 (4.3) | 39 (3.3) |
| **Lack of job control** median (IQR) | | 48.1 (22.2) | 44.4 (25.9) | 40.7 (25.9) | 33.3 (33.3) | 48.1 (29.6) | 40.7 (29.6) | 37.0 (25.9) | 25.9 (25.9) |
| **Perceived discrimination** n (%) | | 330 (27.8) | 330 (25.1) | 365 (24.3) | 236 (17.9) | 208 (35.4) | 421 (31.0) | 381 (28.1) | 182 (14.8) |
| **Negative life event** n (%) | | 753 (36.7) | 912 (30.8) | 1058 (29.7) | 877 (33.7) | 407 (30.7) | 914 (32.8) | 876 (35.4) | 735 (40.3) |
|  |  |  |  |  |  |  |  |  |  |

**Electronic Supplementary Material 2 (table).** Contribution of financial stress to the relative index of inequality for education on lnBRS, lnSDNN and lnRMSSD, after exclusion of individuals taking antihypertensive medication.

|  |  |  |  |  |  |  |  |
| --- | --- | --- | --- | --- | --- | --- | --- |
|  |  | **Indirect effect** | | **Total effect** | | **Proportion mediated** | |
|  |  | *β (CI)* | *p value* | *β (CI)* | *p value* | *proportion* (CI)* | *p value* |
| **Women** | lnBRS^a^ | 0.006 (-0.007, 0.018) | 0.403 | 0.111 (0.047, 0.174) | <0.001 | 0.050 (-0.070, 0.170) | 0.416 |
|  | lnSDNN^b^ | 0.015 (0.004, 0.026) | 0.007 | 0.088 (0.035, 0.142) | <0.001 | 0.173 (0.010, 0.335) | 0.037 |
|  | lnRMSSD^c^ | 0.009 (-0.004, 0.022) | 0.168 | 0.024 (-0.040, 0.087) | 0.462 | 0.382 (-0.771, 1.535) | 0.516 |
| **Men** | lnBRS | 0.000 (-0.014, 0.014) | 0.996 | 0.136 (0.075, 0.198) | <0.001 | 0.000 (-0.102, 0.103) | 0.996 |
|  | lnSDNN | -0.002 (-0.014, 0.010) | 0.727 | 0.119 (0.067, 0.170) | <0.001 | -0.018 (-0.118, 0.082) | 0.728 |
|  | lnRMSSD | -0.009 (-0.023, 0.006) | 0.228 | 0.004 (-0.060, 0.068) | 0.910 | -2.416 (-44.580, 39.749) | 0.911 |
|  |  |  |  |  |  |  |  |

Analyses repeated in a sample, excluding individuals using antihypertensive medication (β blockers, calcium channel blockers and renin-angiotensin system blockers). a lnBRS, natural log of the baroreflex sensitivity; b LnSDNN, natural log of the standard deviation of the NN interval; c lnRMSSD = natural log of the root mean square of successive differences between normal heartbeats; CI, confidence interval.

**Electronic Supplementary Material 3 (table).** Contribution of financial stress to the relative index of inequality for education on lnBRS, lnSDNN and lnRMSSD in women.

|  |  |  |  |  |  |  |  |
| --- | --- | --- | --- | --- | --- | --- | --- |
| **Women** | | | | | | | |
|  |  | **lnBRS**^a^ | | **lnSDNN**^b^ | | **lnRMSSD**^c^ | |
|  |  | *β (CI)* | *p value* | *β (CI)* | *p value* | *β (CI)* | *p value* |
| Finstress^d^ 1^e^ | Indirect^h^ | 0.002 (-0.002, 0.010) | 0.300 | 0.004 (0.000, 0.010) | 0.060 | 0.003 (-0.001, 0.010) | 0.140 |
|  | Total^i^ | 0.172 (0.105, 0.240) | <0.001 | 0.088 (0.023, 0.150) | 0.020 | 0.029 (-0.042, 0.090) | 0.340 |
| Finstress 2^f^ | Indirect | 0.013 (-0.003, 0.030) | 0.140 | 0.018 (0.004, 0.030) | <0.001 | 0.009 (-0.005, 0.020) | 0.260 |
|  | Total | 0.222 (0.147, 0.310) | <0.001 | 0.134 (0.066, 0.200) | <0.001 | 0.090 (0.022, 0.160) | <0.001 |
| Finstress 3^g^ | Indirect | 0.024 (0.004, 0.040) | <0.001 | 0.025 (0.008, 0.040) | <0.001 | 0.019 (0.001, 0.040) | 0.040 |
|  | Total | 0.183 (0.112, 0.270) | <0.001 | 0.166 (0.103, 0.230) | <0.001 | 0.100 (0.013, 0.220) | 0.020 |
|  |  |  |  |  |  |  |  |

lnBRS = natural log of baroreflex sensitivity, lnSDNN = natural log of the standard deviation of the NN interval, lnRMSSD = natural log of the root mean square of successive differences between normal heartbeats; d Finstress = financial stress, e Finstress 1 = second lowest category of financial stress compared to the lowest category of financial stress, f finstress 2 = second highest category compared to the lowest category and g finstress 3 = highest category compared to the lowest category; h Indirect = the indirect effect of education on lnBRS, lnSDNN or lnRMSSD via financial stress, i Total = the total effect (direct + indirect effects) of education on lnBRS, lnSDNN or lnRMSSD,

**Electronic Supplementary Material 4 (table).** Contribution of financial stress to the relative index of inequality for education on lnBRS, lnSDNN and lnRMSSD in men.

|  |  |  |  |  |  |  |  |
| --- | --- | --- | --- | --- | --- | --- | --- |
| **Men** | | | | | | | |
|  |  | **lnBRS**^a^ | | **lnSDNN**^b^ | | **lnRMSSD**^c^ | |
|  |  | *β (CI)* | *p value* | *β (CI)* | *p value* | *β (CI)* | *p value* |
| Finstress^d^ 1^e^ | Indirect^h^ | -0.003 (-0.011, 0.000) | 0.320 | -0.001 (-0.009, 0.000) | 0.640 | -0.005 (-0.013, 0.000) | 0.060 |
|  | Total^i^ | 0.173 (0.106, 0.230) | <0.001 | 0.107 (0.044, 0.170) | <0.001 | 0.020 (-0.061, 0.090) | 0.580 |
| Finstress 2^f^ | Indirect | -0.006 (-0.022, 0.010) | 0.400 | -0.006 (-0.019, 0.010) | 0.400 | -0.013 (-0.028, 0.000) | 0.060 |
|  | Total | 0.193 (0.114, 0.270) | <0.001 | 0.161 (0.089, 0.230) | <0.001 | 0.102 (0.020, 0.180) | <0.001 |
| Finstress 3^g^ | Indirect | 0.012 (-0.006, 0.030) | 0.140 | 0.005 (-0.012 , 0.020) | 0.560 | 0.003 (-0.011, 0.020) | 0.860 |
|  | Total | 0.163 (0.081, 0.260) | <0.001 | 0.150 (0.083, 0.220) | <0.001 | 0.062 (-0.016, 0.150) | 0.200 |
|  |  |  |  |  |  |  |  |

lnBRS = natural log of baroreflex sensitivity, lnSDNN = natural log of the standard deviation of the NN interval, lnRMSSD = natural log of the root mean square of successive differences between normal heartbeats; d Finstress = financial stress, e Finstress 1 = second lowest category of financial stress compared to the lowest category of financial stress, f finstress 2 = second highest category compared to the lowest category and g finstress 3 = highest category compared to the lowest category; h Indirect = the indirect effect of education on lnBRS, lnSDNN or lnRMSSD via financial stress, i Total = the total effect (direct + indirect effects) of education on lnBRS, lnSDNN or lnRMSSD,
